# Schwannoma Localized Retroperitoneally in a 14-Year-Old Boy

**DOI:** 10.1155/2016/1210874

**Published:** 2016-12-18

**Authors:** Hasan Cayirli, Halil Ibrahim Tanriverdi, Ali Aykan Ozguven, Cuneyt Gunsar, Betul Ersoy, Ali Riza Kandiloglu

**Affiliations:** ^1^School of Medicine, Department of Pediatric Surgery, Celal Bayar University, Manisa, Turkey; ^2^School of Medicine, Department of Pediatric Oncology, Celal Bayar University, Manisa, Turkey; ^3^School of Medicine, Department of Pediatric Endocrinology, Celal Bayar University, Manisa, Turkey; ^4^School of Medicine, Department of Pathology, Celal Bayar University, Manisa, Turkey

## Abstract

Schwannomas usually occur in adults being between the second and fifth decades, and such neoplasms are extremely rare in a pediatric population. In addition, they are not normally found in the retroperitoneal region. Here, we present a pediatric case of a retroperitoneal schwannoma in an adrenal location where the tumor was not able to be preoperatively differentiated from other benign or malign adrenal gland tumors. In our opinion, this tumor can be included in the differential diagnosis of a nonfunctioning retroperitoneal adrenal mass in children.

## 1. Introduction

Schwannomas are usually seen in adults between the second and fifth decades of life [1]. They are very rare in a pediatric population. The retroperitoneal region is an unusual location for schwannomas, with approximately 0.5–5% of all cases of schwannoma being retroperitoneal [2, 3]. Only patients with von Recklinghausen's disease have a stronger correlation with retroperitoneal schwannomas [2]. Retroperitoneal schwannomas are, on the whole, mostly benign but malignant tumors have also been reported [1].

Here, we present a pediatric case of a retroperitoneal schwannoma in an adrenal location where the tumor was unable to be preoperatively differentiated from other benign or malign adrenal gland tumors.

## 2. Case Report

A 14-year-old boy was referred to our institution with a retroperitoneal left adrenal mass that was detected on abdominal ultrasound during an investigation into complaints of intermittent abdominal pain and vomiting for about one year. A physical examination of his abdomen found no palpable mass but revealed tenderness in the left lower quadrant. The patient's medical history revealed rectal and intravesical ulcerations medicated 5 years previously and a tonsillectomy 1 year previously, and he had spotty skin pigmentations dissimilar to café au lait spots.

**Figure 1:**
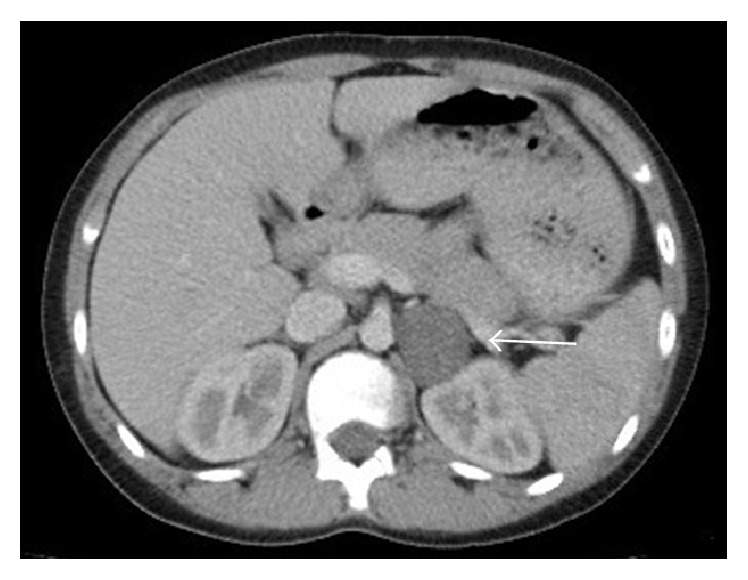
A 32 × 40 mm homogeneous mass (white arrow) in the left adrenal region showing a regular contour on computed tomography.

**Figure 2:**
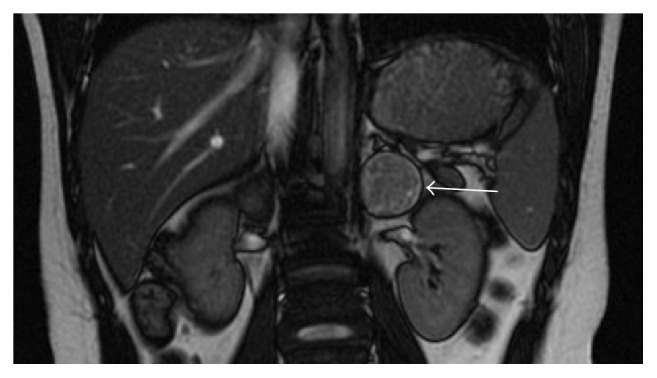
A solid mass (white arrow) 35 × 37 mm in diameter was identified in the left adrenal region in magnetic resonance imaging.

The patient's routine blood tests were normal and peripheral blood smear, lactate dehydrogenase, ferritin, neuron-specific enolase, adrenocorticotropic hormone (ACTH), cortisol and urine vanillylmandelic acid (VMA), homovanillic acid, and metanephrine values were within the normal ranges. No pathological findings were found in the results of a chest X-ray, bone marrow aspiration, and biopsy. Ultrasonography demonstrated a hypoechoic 30 × 37 mm solid lesion close to the medial upper pole of the left kidney causing a change in kidney contour. A dynamic computed tomography (CT) scan of the abdomen confirmed the ultrasound findings and revealed a 32 × 40 mm homogeneous mass in the left adrenal region with a regular contour (Figure 1). Abdominal magnetic resonance imaging was performed to further characterize the lesion and a solid mass 35 × 37 mm in diameter was detected in the left adrenal region (Figure 2). The mass was well defined and did not show any signs of infiltration into the surrounding tissues, indicating a benign process such as an oil-poor adenoma. The retroperitoneal lesion in our patient had regular borders without any sign of adjacent organ invasion which, radiologically, was highly suggestive of a benign lesion.

**Figure 3:**
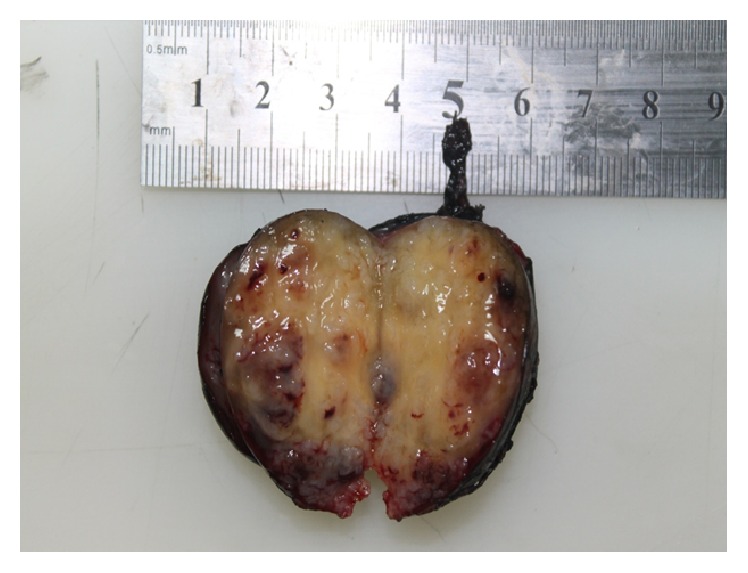
Macroscopic appearance of the tumor. The tumor capsule is smooth and the tumor contains yellow and gelatinous areas as well as hemorrhages.

**Figure 4:**
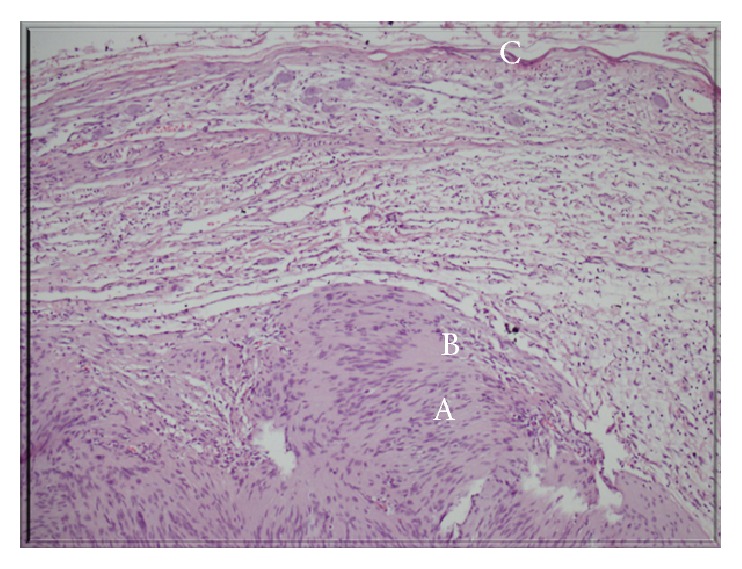
The tumor's microscopic sections: the fibrous capsule (C) and Antoni A areas where the cells showed a Palisade sequence (A) and hypocellular/loose Antoni B areas (B) (Hematoxylin and Eosin, ×100).

During surgery, the abdomen was opened in a transperitoneal way via a left subcostal incision; entering through the bursa omentalis, the pancreas was deviated cranially and a 4.5 cm mass that was immobile, stiff, and adherent to the surrounding tissue was completely excised, including part of the adrenal gland. Histopathological examination showed features of a benign schwannoma. The mass was solid and had smooth surface capsule. There were yellow and gelatinous areas as well as hemorrhages (Figure 3). In the microscopic evaluation, there were hypocellular and loose areas (Antoni B) under the fibrous capsule and there were areas where the cells showed a Palisade sequence (Antoni B) (Figure 4). Immunohistochemical analysis of the tumor showed that the Ki-67 proliferation index was low (5%) and that the S-100 stain was diffuse and strongly positive, confirming that the tumor had a peripheral nerve origin.

The clinical course was uneventful and the patient was subsequently discharged on postoperative day 11. During the first year after the operation the patient was controlled using ultrasound and no problems were detected. After this period, the patient was lost to follow-up.

## 3. Discussion

Schwannomas are tumors of the nerve sheaths that have a generally benign nature [1, 4–6]. The affected part of the body generally comprises subcutaneous tissue of the head or neck region or the distal extremities [2, 4, 5]. The retroperitoneal region, in contrast, is not a common location for schwannomas except in patients with von Recklinghausen's disease (5–8% of all cases) [2]. Schwannomas constitute approximately 1–5% of all cases of retroperitoneal tumor. Interestingly, our case showed a retroperitoneal location but the patient did not have von Recklinghausen's disease.

A search of the medical literature reveals that schwannoma cases originating in the adrenal medulla are very rare. Often, retroperitoneal schwannomas, particularly those located in the juxta-adrenal space, cannot be diagnosed preoperatively when they mimic adrenal lesions. In Poland, out of 1,111 adrenal incidentaloma cases that were reviewed, only 2 appeared to show schwannomas [7]. Our case was symptomatic with complaints of abdominal pain and vomiting; therefore, the mass was not diagnosed as an incidentaloma.

The symptoms of a benign schwannoma are usually nonspecific and change according to the location and size of the lesion [1]. Retroperitoneal schwannomas are generally asymptomatic; thus, it is difficult to determine a diagnosis preoperatively [8]. The most common symptoms are abdominal pain and distention [1, 2], while other symptoms include secondary hypertension, hematuria, and renal colic, depending on the location of the lesions [1]. In our case, the patient did not have hypertension or hematuria.

In general, malignant schwannomas are diagnosed in patients who have neurofibromatosis type 1 and neurofibromatosis type 2 [6]. The colon, kidneys, and adjacent viscera are rarely invaded by schwannomas that undergo malignant transformation [2]. Neither the size of the lesion nor the depth of invasion is associated with the possibility of malignancy. In our patient, histopathological examination showed features of a benign schwannoma, although the diameter of the mass was more than 4 cm during the operation and it was adherent to the surrounding tissue.

Other neurogenic tumors such as paraganglioma and pheochromocytoma are considered additional pathologies in the differential diagnosis of retroperitoneal masses [1, 4]. If the mass shows cystic degeneration, retroperitoneal cystic masses such as hematoma and lymphangioma should also be considered [1]. The radiological findings of our patient led us to believe that the diagnosis was an oil-poor adenoma, but both benign and malignant tumors must be considered preoperatively as a differential diagnosis.

Hormone-releasing tumors must be excluded by the clinician if an adrenal lesion is present, particularly pheochromocytoma. Because hemorrhage, infection, and seeding of the tumor cells are known risks and are lethal if there is a pheochromocytoma, biopsy is not recommended for diagnosis. Distinguishing among adrenal schwannomas based on diagnostic imaging studies can be difficult [4]. The standard metabolic examination includes serum electrolytes, cortisol, aldosterone, ACTH, 17-ketosteroids and 17-hydroxycorticoids, renin, urinary catecholamines, metanephrine, and VMA. Almost none of the cases show clinical or biochemical evidence of endocrine hormonal activity [4]. We performed the standard metabolic examination for our patient and found no clinical or biochemical evidence of endocrine hormonal activity. However, our patient did not undergo biopsy.

Because malignancy cannot be excluded with analyses performed pre- or perioperatively and because schwannomas are insensitive to radiotherapy and chemotherapy, complete resection of the tumor is recommended [6, 8]. Benign schwannomas have a good prognosis. Recurrence is the most frequent complication, reported in 5–10% of cases, and is mostly related to incomplete excision [6]. Complete resection was performed in our case and the patient experienced no complications during follow-up.

Although Antoni A and Antoni B areas are seen in various ratios in classic schwannoma, varying morphologies have been described, including ancient, cellular, melanotic, plexiform, glandular, and epithelioid subtypes [5]. According to Mohiuddin and Gilliland, ancillary studies have demonstrated that the immunohistochemistry of schwannomas shows strong and diffuse staining for S-100 and that they are typically negative for CD117, desmin, CD34, HMB-45, synaptophysin, chromogranin, cytokeratin, and smooth muscle actin [4]. In our patient, the Ki-67 proliferation index was low and the S-100 stain was diffuse and strongly positive.

In view of the above, we believe that this tumor can be included in the differential diagnosis of a nonfunctioning retroperitoneal adrenal mass in children.
